# A suicide attempt by ingestion of oleander leaves and treatment with digoxin-specific Fab antibody fragments

**DOI:** 10.2478/aiht-2023-74-3752

**Published:** 2023-12-29

**Authors:** Tanja Kovačević, Branka Polić, Tatjana Ćatipović Ardalić, Davor Petrović, Luka Stričević, Maja Rogulj, Joško Markić

**Affiliations:** University Hospital of Split, Department of Paediatrics, Split, Croatia; University of Split School of Medicine, Split, Croatia; University Hospital of Split, Department of Psychiatry, Split, Croatia

**Keywords:** antidotes, common oleander, heart block, *Nerium oleander*, oleandrin poisoning, *Thevetia peruviana*, yellow oleander, antidot, *Nerium oleander*, oleandrin, otrovanje, srčani blok, *Thevetia peruviana*

## Abstract

Natural cardiac glycosides have positive inotropic heart effects but at high, toxic doses they can cause life-threatening cardiac arrhythmias. Here we present the first Croatian case of a 16-year-old girl who attempted suicide by eating dried oleander leaves, which contain natural cardiac glycosides, and her treatment with a specific antidote. The girl presented with an oedema of the uvula indicating local toxicity, severe bradycardia, first-degree atrioventricular block, drowsiness, and vomiting. Having taken her medical history, we started treatment with atropine, intravenous infusion of dextrose-saline solution and gastroprotection, but it was not successful. Then we introduced digoxin-specific Fab antibody fragments and within two hours, the patient's sinus rhythm returned to normal. Cases of self-poisoning with this oleander are common in South-East Asia, because it is often used as a medicinal herb, and digoxin-specific Fab fragments have already been reported as effective antidote against oleander poisoning there. Our case has taught us that it is important to have this drug in the hospital pharmacy both for digitalis and oleander poisoning.

## Abstract (sl)

Prirodni srčani glikozidi imaju pozitivan inotropni učinak na srce. U visokim, toksičnim dozama mogu prouzročiti za život opasne srčane aritmije. Predstavljamo prvi hrvatski slučaj pokušaja samoubojstva 16-godišnje djevojke konzumiranjem oleandrovih suhih listova, koji sadrže prirodne srčane glikozide, i liječenje specifičnim protuotrovom. Djevojka je također imala edem uvule kao znak lokalne toksičnosti. Svi podatci dobiveni su analizom dokumentacije iz medicinskoga kartona. U djevojke se javila značajna bradikardija, atrioventrikularni blok prvog stupnja, pospanost i povraćanje. Početno liječenje atropinom, intravenskom infuzijom fiziološke otopine i 5 % glukoze uz gastroprotekciju nisu bili učinkoviti. Pacijentica je zatim liječena digoksin-specifičnim Fab imunoglobulinskim fragmentima. Unutar dva sata od primjene lijeka srčani se ritam promijenio u normalni sinusni ritam. Ovo je rijedak slučaj pokušaja suicida samootrovanjem na našim prostorima. Slučajevi samootrovanja ovom biljkom česti su u jugoistočnoj Aziji jer se ondje često koristi kao biljni proizvod za samoliječenje. Digoksin specifični Fab fragmenti su protuotrov za predoziranje digoksinom, kao i za akutnu intoksikaciju ovom biljkom. Važno je imati ovaj lijek u bolničkoj ljekarni kako za intoksikaciju digitalisom tako i za otrovanje ovom biljkom.

Digoxin, ouabain, bufalin, and oleandrin are well-known natural cardiac glycosides, which bind to and inhibit the sodium-potassium pump and thus have a positive inotropic effect on the heart muscle (1). They are parts of plant, toad, and mammal tissues (2) of similar chemical structure consisting of a steroid ring, a lactone ring, and a sugar moiety (3) and therefore of similar pharmacological effects.

Oleandrin, as its name suggests, is present in oleander species of the *Apocynaceae* family, including the seeds, roots, leaves, flowers, branches, and stem. It dissolves well in water, alcohol, ether, and chloroform and cannot be inactivated by drying and cooking the leaves (4). Low doses of oleandrin improve heart function, slow heart rate, and act as a mild diuretic. High, toxic doses can cause premature ventricular contraction, junctional rhythm, atrioventricular block, atrial fibrillation, ventricular tachycardia and fibrillation, sinus tachycardia, idioventricular rhythm, His bundle block, sinus arrest, junctional escape rhythm, and atrial flutter (5, 6, 7, 8). Other possible symptoms of oleandrin poisoning are nausea, abdominal pain, vomiting and diarrhoea, visual disturbances, dizziness, muscle weakness, amnesia, confusion, delirium, hyperpyrexia, and hyperkalaemia (2, 9). These effects are the same whether they are caused by the common oleander (*Nerium oleander*) or the yellow oleander (*Thevetia peruviana*). However, their onset and the severity of poisoning depend on the patient, parts of the plant ingested, ingested amount, and the way the poison is prepared (1).

Oleander poisoning is most often accidental in children, pets, or livestock due to ingestion of contaminated food (10, 11, 12), and such cases have been relatively common in South-East Asian countries, where oleander is often used as medicinal herb for slimming, muscle enhancement, erectile dysfunction, epilepsy, psoriasis, herpes, eczema, thyroiditis, and cancer treatment (1, 13, 14, 15). However, suicidal poisoning with oleander tea has also been reported quite frequently in South-East Asia, especially among teenagers and adults, which is not the case with Europe and the USA (1, 14, 16, 17, 18, 19).

To the best of our knowledge, here we present the first such case of suicide attempt with dried oleander leaves in Croatia and describe how we managed the poisoning by resorting to a specific antidote after a failed first attempt with standard treatment.

This study was approved by the Ethics Committee of the University Hospital of Split (approval No. 2181-147/01/06/LJ.Z.-23-02). All data were collected from the patient's medical record, and the patient's mother signed informed consent to publication.

## CASE REPORT

A 16-year-old girl was admitted to the hospital's emergency unit six to seven hours after ingesting 20 dried oleander leaves (*Nerium oleander*), which she had harvested and dried at home over the three preceding months. She presented with cramping abdominal pain, strong nausea, and vomiting. She also complained of shortness of breath and was sweating profusely. Prior to admission, seeing that the patient's uvula had swollen the ambulance medical team administered 20 mg of chloropyramine via intramuscular injection and 40 mg of methylprednisolone and saline via intravenous infusion.

From the emergency unit, she was moved to the paediatric intensive care unit due to irregular and slow heart rate, mild hypotension, and somnolence ten hours after poisoning. She was breathing normally and was afebrile but sweating still. Numerous old scars from self-harming were visible on her limbs. There was no oedema in the oral cavity. The abdomen was diffusely painful on palpation. Her body weight was 47.4 kg, height 171 cm, and BMI 16.21 kg/m^2^, indicating malnourishment. The rest of the physical examination findings were normal.

The first electrocardiography (ECG) and telemetry revealed marked sinus bradycardia at a rate of 30–45 per minute with frequent sinus pauses and maximal RR interval of 2.5 seconds. There was also a mild downsloping ST depression. T waves were inverted in the lower leads.

Laboratory findings were completely normal, including potassium (4.4 mmol/L), sodium (138 mmol/L), chlorine (103 mmol/L), calcium (2.44 mmol/L), phosphorus (0.83 mmol/L), magnesium (0.81 mmol/L), and glucose (5.2 mmol/L). No blood alcohol was detected. The lowest serum concentration of potassium in the days of treatment that followed was 3.6 mmol/L. Urine and blood toxicology revealed caffeine and nicotine metabolites. Due to technical issues, analysis of oleandrin, oleandrigenin, neritaloside or odoroside was not possible, whereas digoxin was not analysed on arrival by omission. Furthermore, activated charcoal was not administered due to significant time lag between poisoning and admission to the emergency unit. In the first 24 hours of hospitalisation, the patient received seven 0.5 mg doses of atropine intravenously in order to raise the heart rate and normalise blood pressure, since the systolic pressure ranged from 70 to 95 mmHg. Besides, the patient was receiving continuous intravenous infusion of 5 % dextrose with saline and gastro-protection with pantoprazole.

Given that the effect of atropine was short-term, that it had to be administered frequently, and that her condition did not significantly improve, we decided to use an antidote and placed an urgent order for digoxin immune Fab (DIGIFab^®^, BTG International Inc., West Conshohocken, PA, USA), which arrived the following day. The first digoxin concentration measured on day two of hospitalisation was 0.5 ng/mL (reference range 0.8–2.0 ng/mL). An empiric dose of ten 400 mg vials of digoxin-specific fab immunoglobulin fragments diluted in saline was administered intravenously over two hours, 28 h after ingestion of oleander leaves. The patient was subfebrile during infusion, but later the temperature returned to normal. The PQ interval normalised and sinus pauses disappeared two hours after the infusion was completed. Her heart rate remained ≥60/min while awake, and blood pressure and consciousness were normal. However, a mild sinus bradycardia was noticed during sleep. As the overall condition improved, save for vomiting and diarrhoea during the first two days, infusion with digoxin-specific Fab antibody fragments was not repeated. The patient was then moved to paediatric psychiatric hospital. At the time of discharge, she was symptom-free and had a normal ECG.

The girl had had suicidal ideations and was planning this for almost a year. In her own words, she was “fed up with life” and “didn’t see the future”. Psychiatric observation during hospitalisation and outpatient follow-up confirmed a number of cognitive and behavioural risk factors (earlier suicidal considerations and ideations, self-harm and impulsive aggression), developed depressive disorder, and unfavourable family environment involving parental psychopathology and dysfunctional partnership. According to the psychodynamic concept, the patient had an intrapsychic conflict of non-acceptance and repulsion for the female body and sexuality; she identified maturity for partnership/relationship and echoed her mother's controlling tendencies opposed to the evolutionary need for independence. The trigger for the girl's self-aggression was the deterioration of mother's health and her hospitalisation shortly before the girl's suicide attempt in response to growing anxiety, disappointment, and the sense of helplessness. Self-loathing and aggression stemmed from the fundamental feeling of insecurity and guilt. Further treatment included concerted effort of paediatricians, child psychiatrists, and social workers and involved psychoeducational work with her and her mother. Since then, she has been followed by a psychiatrist once a month. She still skips school often and struggles with the grades, but her mood is stable, and communication in the family has much improved. She does not hurt herself any more nor does she show other signs of auto-aggression.

## DISCUSSION

Persons who intentionally poison themselves most often choose pharmaceutical or chemical products. The use of herbal poisons is extremely rare, especially in the paediatric population of Europe. An exception is the report by Azzalini et al. (20) about self-poisoning by voluntarily ingesting oleander leaves in Italy.

Besides systemic toxicities, local corrosive findings can be present when the plant is chewed, swallowed, or drunk. Taskin et al. (21) describe a case of oropharyngeal oedema, uvular congestion, and mucosal necrosis after chewing the leaves of the common oleander. In our case, the teenage girl had oedematous uvula, which is why she received chloropyramine and methylprednisolone on the first examination by the emergency team at home. Subsequent examinations showed no signs of oedema. We assume that our patient had milder local signs of toxicity because the ingested oleander leaves were dry, unlike the Taskin's case, in which the patient chewed a fresh leaf.

Our treatment choice and dosing regimen of digoxin-specific antibody fragments was largely based on the 2005 report by Camphausen et al. (22) about safe and effective treatment of a seven-year-old girl and on the randomised controlled study by Eddleston et al. (23), who investigated clinical effects of anti-digoxin Fab in cases of serious oleander-induced arrhythmias, as well as on other studies reporting its effectiveness and safety of use (17, 24, 25). Digoxin immune Fab is effective in reverting life-threating cardiac arrhythmias caused by digitalis poisoning. It has a superior affinity for digoxin than has digoxin for the sodium-potassium pump. It binds to digoxin molecules and reduces its free concentrations, lowering thus its cardio-toxic effects (26). According to the healthcare data (17), it is also effective in the treatment of life-threatening conditions of acute poisoning with the common and yellow oleander.

In standard care, the toxic effects of oleandrin are treated symptomatically. As the bradycardia caused by it is of the vagal origin, the usual treatment choice is atropine, whereas severe cases are managed with temporary cardiac pacing. In our case, we started with atropine to treat bradycardia and first-degree AV block, but the response was not satisfactory. Electrolyte stability and adequate hydration were maintained without difficulties. In order to control severe vomiting, we administered pantoprazole instead of an antiemetic such as ondansetron, because this drug can worsen cardiac arrhythmias.

Digoxin blood level in our patient on the second day of hospitalisation was low, in fact, within the therapeutic range. However, we were aware that digoxin blood levels found in patients poisoned with herbs that contain cardiac glycosides do not necessarily correlate with the severity of poisoning, as oleander alone has more than 30 different cardiac glycosides (2). This is why dosing with digoxin-specific antibody fragments should not rely on digoxin or digitoxin blood levels (22, 25). Oleandrin poisoning calls for higher doses of digoxin-specific antibody fragments, because the antibody has lower binding affinity for natural cardiac glycosides (1). In fact, the clinical study by Eddleston et al. (23) showed more frequent conversion to sinus rhythm or reversal of hyperkalaemia at higher, 800–1600 mg doses (20 to 40 vials) than with lower doses of 400 mg (10 vials). Side effects such as pruritus, angioedema, or bronchospasm occurred in almost a quarter of patients receiving digoxin-specific Fab fragments. Having all this in mind, we administered the 400 mg dose (10 vials) to our patient minding that it is given slowly by infusion over two hours instead of the usual 20 min, since this was our first such treatment. Our patient had no severe side effects and clinical improvement was immediately evident.

## CONCLUSION

Self-poisoning with oleander leaves could become a serious concern, especially among teenagers, even though it has not been common in the western countries, unlike South-East Asia. One of our distinct findings is that oleander poisoning in our patient caused a local corrosive effect on the oropharynx in addition to systemic toxic effects.

It is important to be prepared for the most unusual cases of poisoning in adolescents nowadays, and digoxin immune Fab is a safe and effective antidote for oleander poisoning. Therefore, we believe that paediatric intensive care units should have this antidote available in the hospital pharmacy.
